# Artificial spatiotemporal touch inputs reveal complementary decoding in neocortical neurons

**DOI:** 10.1038/srep45898

**Published:** 2017-04-04

**Authors:** Calogero M. Oddo, Alberto Mazzoni, Anton Spanne, Jonas M. D. Enander, Hannes Mogensen, Fredrik Bengtsson, Domenico Camboni, Silvestro Micera, Henrik Jörntell

**Affiliations:** 1 The BioRobotics Institute, Scuola Superiore Sant’Anna, Pisa, Italy; 2 Neural Basis of Sensorimotor Control, Department of Experimental Medical Science, Lund University, Lund, Sweden; 3 Bertarelli Foundation Chair in Translational NeuroEngineering, Center for Neuroprosthetics and Institute of Bioengineering, School of Engineering, École Polytechnique Federale de Lausanne, Lausanne, Switzerland

## Abstract

Investigations of the mechanisms of touch perception and decoding has been hampered by difficulties in achieving invariant patterns of skin sensor activation. To obtain reproducible spatiotemporal patterns of activation of sensory afferents, we used an artificial fingertip equipped with an array of neuromorphic sensors. The artificial fingertip was used to transduce real-world haptic stimuli into spatiotemporal patterns of spikes. These spike patterns were delivered to the skin afferents of the second digit of rats via an array of stimulation electrodes. Combined with low-noise intra- and extracellular recordings from neocortical neurons *in vivo*, this approach provided a previously inaccessible high resolution analysis of the representation of tactile information in the neocortical neuronal circuitry. The results indicate high information content in individual neurons and reveal multiple novel neuronal tactile coding features such as heterogeneous and complementary spatiotemporal input selectivity also between neighboring neurons. Such neuronal heterogeneity and complementariness can potentially support a very high decoding capacity in a limited population of neurons. Our results also indicate a potential neuroprosthetic approach to communicate with the brain at a very high resolution and provide a potential novel solution for evaluating the degree or state of neurological disease in animal models.

Haptic skin-object interactions necessary for touch perception generate spatiotemporal patterns of activation across multiple tactile skin sensors. When different kinds of objects interact with the same part of the skin, they can be expected to generate different such spatiotemporal patterns^1^,^2^, which form part of the brain’s perception of the skin-object interaction. Even though the interpretation of the ‘what’ such inputs represent is distributed across a large number of neocortical neurons, the information must to some extent be expressed also at the level of individual neurons. In other words, at least the bits and pieces of the population-level information should be represented in the single neuron. It has commonly been observed that skin-object interactions with different kinds of objects and shapes generate different neural responses^3^,^4^,^5^, but the precision by which neocortical neurons can distinguish specific spatiotemporal patterns of tactile afferent input (i.e. identifying the ‘what’ of the input) from the same part of the skin has to our knowledge not previously been estimated.

Analysis and quantification of the specific decoding of tactile information in a cortical neuron requires the repeated presentation of reproducible spatiotemporal patterns of skin sensor afferent activation. But the mechanical activation of the skin^6^, the mechanotransduction into an electrical receptor potential and the spike generation from that receptor potential in the tactile afferent axon^7^ are potential sources of noise that could result in variability in the spatiotemporal pattern from one trial to another^2^,^8^,^9^. In addition, even small shifts in the position of a mechanical stimulus would result in that the spatiotemporal pattern of skin sensor activation shifts across the recruited population of sensors, which at the level of the decoding in the neuronal circuitry corresponds to a different task, even though the perception may be essentially the same. To overcome these difficulties, our aim was to by-pass the potentially variable steps that come with the mechanical skin sensor activation, so that the decoding capacity of central neurons could be estimated in relative isolation. Using direct electrical interfacing with the distal nerve fibers innervating the skin sensors, it becomes possible to investigate the neuronal decoding of a ‘frozen state’ of sensor activation, isolated from the uncertainty in the mechanotransduction step. With this approach it becomes possible to repeat multiple spatiotemporal patterns a high number of times in a random order even within the lifetime of an intracellular neuronal recording. The findings obtained using direct neural stimulation also have potential translational importance for limb neuroprosthetics. The method was first introduced in human studies, using percutaneous electrical microstimulation of tactile afferents using a single channel electrode^10^,^11^. Using long-term peripheral nerve implants with temporally patterned direct electrical activation of different portions of a nerve trunk, diversified and meaningful perceptions of touch can be achieved^12^,^13^,^14^,^15^,^16^, which indicates that the brain can actively engage in the processing of electrically induced tactile afferent input.

In auditory and visual cortical systems, ‘natural scenes’ of sensory input have been shown to evoke very different cortical responses compared to those evoked by simplified inputs^17^,^18^. This may indicate that the circuitry structure is adapted to some general features in the spatiotemporal patterns of sensor activation that may occur during natural behavior, and that this circuitry structure constrains the way the individual neurons in the network are activated. Following these considerations, we wanted as far as possible to achieve naturalistic, rather than arbitrarily designed, patterns of electrical skin sensor activation. For this purpose, we employed a biomimetic artificial fingertip equipped with an array of neuromorphic tactile sensors that generated spatiotemporal patterns of skin sensor output in response to mechanical touch or indentation with probes characterized by different curvature. The spatiotemporal patterns of spike output generated from this system during dynamic touch were delivered as electrical stimuli to the distal tactile afferents from the volar side of the second digit of the rat forepaw. With this approach, we hoped to mimic at least the general envelope of the overall temporal modulation of activation in the local population of skin sensors as it may occur in a conceivable natural touch. In this way, the spatiotemporal pattern of skin sensor activation that we delivered may be better tuned to the space of possible patterns of input that the circuitry of the adult animal may have been adapted to. In order to allow well-controlled, repeatable stimulus delivery and long-term *in vivo* whole cell patch clamp recordings from neocortical neurons in the absence of top-down modulations^19^ we used anesthetized rats. We find that the decoding performance of individual neurons can be much higher than previously described and that there is a complementarity in their response profiles that provides for a very high decoding capacity even in small groups of neurons.

## Results

**Figure 1:**
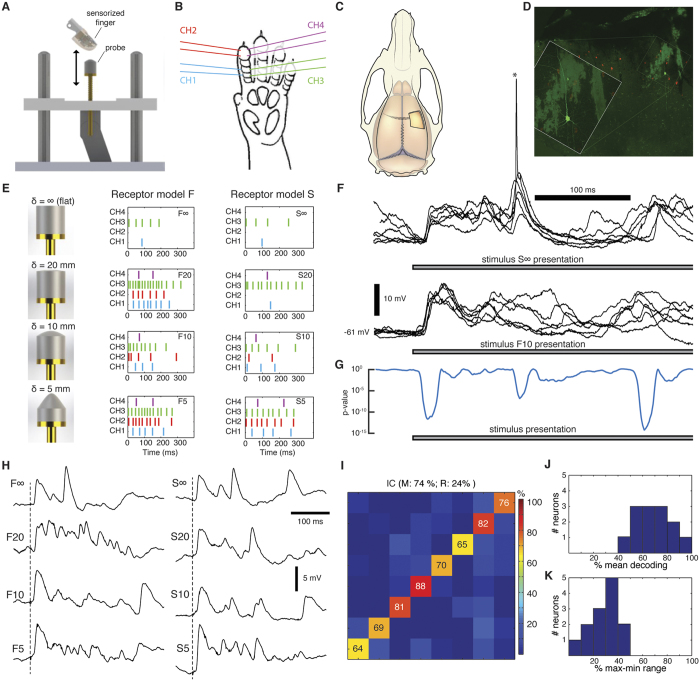
Synthesis of tactile patterns and intracellular responses *in vivo*. (**A**) Shapes with different curvatures were moved against an artificial fingertip using a cyclic motion. The artificial fingertip was equipped with an array of four neuromorphic sensors that generated spatiotemporal spike output patterns that were electrically delivered to the skin of the rat paw (Supplementary Fig. S1). (**B**) The location of the pairs of intracutaneous needle electrodes (channels) delivering the generated spike output patterns. (**C**) Illustration of approximate recording location (in the S1 digit region). (**D**) Sample morphology of a recorded neuron, identified as a layer III pyramid based on morphology and depth. Red staining is parvalbumin immunopositive neurons. (**E**) From left to right: the four types of probe shapes (Supplementary Fig. S1) used; the corresponding plots of the spike output patterns, equalling the spatiotemporal patterns of elementary current pulses delivered to the skin of the rat, obtained with the receptor model F; corresponding plots of the spike output patterns obtained with the receptor model S. (**F**) Superimposed raw intracellular responses evoked by two different stimulation patterns. One neuronal spike (asterisk) is included for display. (**G**) The Welch student’s t-test dissimilarity measure displayed as p-values, comparing the responses evoked by the two stimulation patterns illustrated in (**F**). (**H**) Average intracellular responses of the neuron in (**D**) for each stimulation pattern in (**E**). (**I**) Confusion matrix of the PCA results of the decoding of the different spatiotemporal stimulation patterns for the illustrated neuron. ‘M’ indicates the mean decoding, and ‘R’ the range of decoding (max-min). (**J,K**) Mean and range of decoding for all intracellular recordings.

Our aim was to study the decoding capacity of central neurons in isolation, separated from noise arising from mechanical interfacing with the skin and from intrinsic skin sensor noise (even highly controlled repeated activation of a single mechanical stimulus yields a range of variability in the spiking of the primary sensor afferents of 2 ms on average, with up to 8 ms jitter observed in the spike trains and even missing one or two spikes in each repeated run of a given touch condition^9^). For this purpose we used electrical stimulation of four skin sites (channels) of the volar side of digit 2 of the rat (a skin surface that through the lifetime of the animal would have been activated by a vast array of inputs via touch, locomotion and other motor activities). Using a low stimulation intensity of about 2.5 times threshold for primary afferent activation (Methods), such stimulation activates a limited number of tactile afferents (and no nociceptive afferents whose recruitment starts at 4 times threshold^20^) with a high temporal precision and reliability^21^. In order to generate spatiotemporal patterns of skin sensor activation with overall similarities with patterns that could be evoked under natural conditions such as active touch, we used an artificial fingertip equipped with an array of four separate neuromorphic sensors (Fig. 1A and Supplementary Fig. S1). These neuromorphic sensor have activation properties that are similar to biological tactile sensors for the type of stimuli we used here (Supplementary Fig. S2) as well as other types of stimuli^12^. The spatiotemporal patterns of electrical skin site stimulation to be used with the animal were first obtained from the artificial fingertip by moving it against probes of different shapes (Fig. 1A,B and Supplementary Fig. S1). Out of the many spatiotemporal spike output patterns recorded from the neuromorphic sensors, we selected two patterns for each of the four indentation probes used, to obtain a total of eight richly resolvable spatiotemporal patterns (Supplementary Fig. S1). The comparably high resolvability of the eight patterns used was an important component in our subsequent evaluation of the decoding performance of the neocortical neurons. These eight spatiotemporal patterns of multi-channel electrical skin site activation were used throughout the experimental series (Fig. 1E).

The responses of neocortical neurons to the repeated applications of these spatiotemporal patterns of electrical skin site stimulation were recorded in the paw region of the primary somatosensory cortex (Fig. 1C) using the whole cell patch clamp (N = 14 neurons) or loose-patch, cell-attached (N = 82 neurons) recording techniques. We morphologically recovered 11 neurons recorded in the whole cell mode (Fig. 1D), of which all were pyramidal neurons at layers II–V. Of the 82 cell-attached recordings, 12 neurons had a narrower spike width than the others and could be classified as putative interneurons according to Luczak, *et al*.^17^, but these neurons did not stand out from the other neurons in terms of the response patterns described below and are hence not indicated separately.

### Analysis of intracellular responses

With the whole cell patch clamp method, we recorded the intracellular membrane potential responses resulting from the synaptic inputs evoked by the spatiotemporal skin stimulation patterns (Fig. 1E,F). For each neuron, we first made pairwise comparisons of the responses evoked by two different stimulation patterns (Fig. 1F,G). The average intracellular membrane potential responses were typically unique for each stimulus pattern (Fig. 1H), so for each neuron we made such pairwise comparisons across all pairs of stimulation patterns available (N = 27). For the example cell illustrated in Fig. 1F–H the fraction of comparisons yielding statistically significant differences (KS-test) between pre- and poststimulus times was 93%, which indicates that the synaptic inputs to the cell could be used to separate almost all of the stimulation patterns. Across the population of neurons recorded in the whole cell mode (N = 14), 68+/−20% of the patterns could be separated in these pairwise comparisons, with 4 neurons separating the inputs in more than 90% of the comparisons. Hence, the evoked intracellular responses were relatively specific to the precise stimulation pattern used. In addition to these pairwise comparisons, we also performed a principal component analysis (PCA) of the intracellular responses, where the decoding accuracy for each stimulation pattern could be analysed in relation to the decoding accuracy for all the other patterns. The mean decoding for the sample neuron, according to the PCA, was 74% across all of the eight patterns whereas the decoding of the individual stimulation patterns varied within a relatively narrow range as indicated in the confusion matrix in Fig. 1I. For the population of neurons, the average mean decoding according to the PCA was 69+/−18% (Fig. 1J) with a variable range of decoding across the different stimulation patterns (Fig. 1K).

### Analysis of spike responses in individual neurons

**Figure 2:**
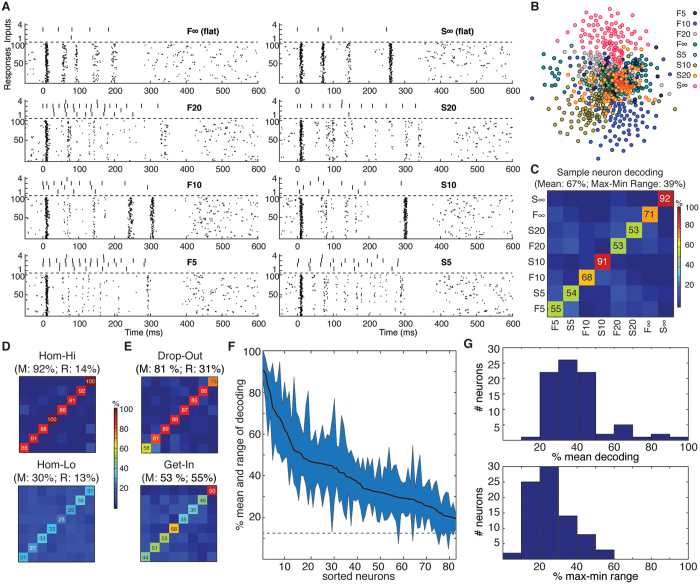
Single neuron decoding for spike output. (**A**) Raster plots of spike responses of a sample neuron to the stimulation patterns as indicated. (**B**) Cluster analysis of the same spike responses. The response to each stimulus presentation is shown color-coded. (**C**) Confusion matrix of the decoding performance for the neuron illustrated in (**A**,**B**) (see Supplementary Fig. S3 for illustration of this method). The title indicates the mean and the range of the decoding across the eight stimulation patterns. Numbers inside the matrix indicate the decoding rates for the individual stimulation patterns. (**D**) Example confusion matrices for two neurons with homogeneous decoding, one with high mean decoding and one with low mean decoding. (**E**) Example confusion matrices for two neurons with heterogeneous decoding. Top, example confusion matrix with ‘drop-out’ decoding. Bottom, confusion matrix for a ‘get-in’ neuron with selective decoding. (**F**) Mean and range of decoding across the population of neurons. The horizontal dashed line indicates chance level. (**G**) Distribution, over the population of neurons, of the mean decoding (top) and of the range of decoding (bottom).

Although the intracellular analysis reveals the input information to the neuron, for the network of the brain the more relevant signal is the resulting neuronal spike output, which is the signal that is being processed by the neuronal network. As there seems to be no simple, direct relationship between the intracellular signal and the resulting pattern of spike output in central neurons^22^,^23^, the spike output needed to be analyzed as well. To analyse the spike output, we used cell-attached recordings. In these recordings, spike responses were in many neurons reliable, precise and input-specific (Fig. 2A) and even the responses to single stimulus presentations could at least for some stimulation patterns form relatively reliable clusters (Fig. 2B). As for the intracellular signals (Fig. 1I–K), the spike responses of individual neurons (N = 82) were also processed via PCA and kNN decoding (Supplementary Fig. S3) to evaluate the precision by which the responses to repeated stimulus presentations could be used to decode the identity of the stimulation pattern applied (Fig. 2C). In the illustrated example (Fig. 2A–C), the neuron was able to segregate the eight stimulation patterns with a mean accuracy of 68%. For one of the stimulation patterns, the decoding performance was as high as 92% (pattern S∞ in Fig. 2C).

**Figure 3:**
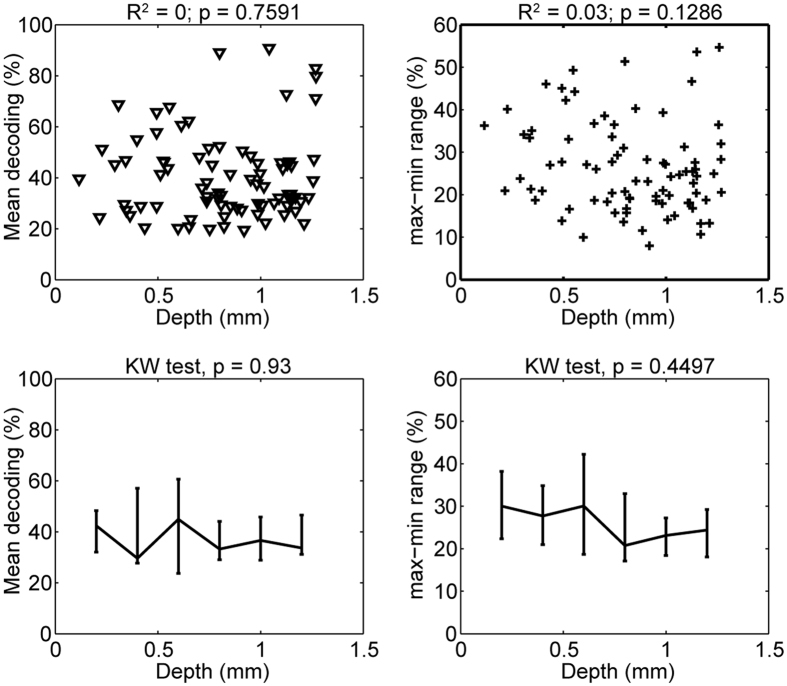
Relationship between neuronal depth and decoding. Lack of relationship between recording depth and decoding (left) and between recording depth and decoding heterogeneity (right). Bottom, corresponding box plots (median and interquartile range) with recording depths pooled in steps of 200 μm. Even though precise laminar information and neuronal type identity was not a focus of our study, the absence of relationship between decoding and recording depth strongly suggested (Pearson linear correlation test and Kruskal Walis test, see text) that there was no relationship between decoding performance and laminar location.

Overall, the top performing neurons had a decoding performance as high as 80–90% (Fig. 2D, top), while most neurons had a mean decoding performance of 20–50% (Fig. 2D, bottom). But the PCA analysis also indicated that in the population of neurons, there was heterogeneity with respect to the decoding performance across the stimulation patterns. Some neurons showed specificity in the sense that the identification rate of at least one stimulation pattern was at least 20% units lower than the mean decoding performance (‘drop-out’, N = 12, Fig. 2E top). There were also neurons with highly specific decoding in the sense that the top decoded stimulation pattern had identification rate at least 20% units higher than the mean decoding performance (‘get-in’, N = 9, Fig. 2E bottom). The mean and range of decoding of the spike output varied across the population (Fig. 2F) (Supplementary Fig. S4 reports the corresponding information content for all the neurons). The distributions of the mean (Fig. 2G, top) and the range (Fig. 2G, bottom) of the decoding performance were compatible with a lognormal fitting (p = 0.46 and p = 0.95, respectively, one-sample KS test)^24^. Neither the mean nor the range of the decoding depended significantly on the depth at which the neuron was recorded (Pearson linear correlation test: p > 0.1 both variables; Kruskal Wallis test, p > 0.1) (Fig. 3). Notably, the mean decoding (Fig. 2G, top) was substantially lower than for the intracellular signals (Fig. 1I).

**Figure 4:**
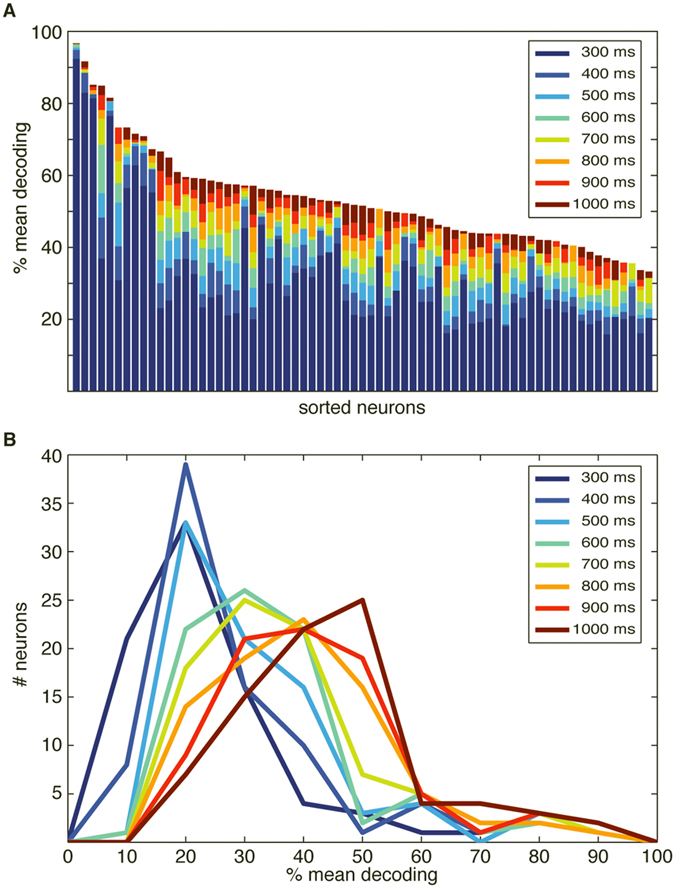
Information content in late response components. (**A**) Dependence of the mean decoding performance (accuracy) on the extent of the time window included in the analysis. Note that none of the stimulation patterns lasted for more than 340 ms, yet in almost all neurons the information increased all the way up to 1000 ms poststimulus time window. Interestingly, neurons also differed widely with respect to the increase in decoding accuracy obtained when the time window was expanded. Only neurons with a decoding above 30% at 1000 ms (N = 69) are included in the display. (**B**) Distribution of the mean decoding performance across the neurons as a function of the time integration window.

The decoding performance of the neurons depended on the interval over which the spike response was integrated, with some neurons allowing very high decoding with short integration windows (Fig. 4). Remarkably, for many other neurons the decoding level slightly increased even up to 1000 ms after the onset of the stimulation (Fig. 4B). As the longest duration of any of the spatiotemporal input patterns was 340 ms, the stimulation patterns hence produced organized activity in the neuronal networks long after the stimulation had ceased.

### Responses evoked by stimulation of single channels

As complementary information to the results above using complex patterns of input, we also analysed responses evoked by simpler stimuli, specifically stimulation of the separate skin input channels with one or two pulses. Notably, although all of our neurons responded to the complex patterns, only 57 out of our 82 neurons had a measurable response to single pulse for every channel. In this group of 57 neurons, we first asked if the neurons could decode whether the input was a single pulse or a double pulse, where the second pulse occurred 300 ms after the first (analysis time window limited to 450 ms in this case). Across each of the 4 stimulation channels (228 comparisons) the decoding was 79.3%, where 7 neurons with strong responses to single pulse stimulation could separate single from double pulse stimulation for at least one channel with 100% precision. In the next step, we asked whether the neurons could tell which of the 4 channels that were stimulated, and whether that channel was stimulated once or twice. Most of the neurons performed well on this task, with a mean decoding across the population of 43% and information content of 1.66 bits (out of 3 bits maximum) (Supplementary Fig. S5).

### Complementary neuronal response patterns allow for co-operative decoding

**Figure 5:**
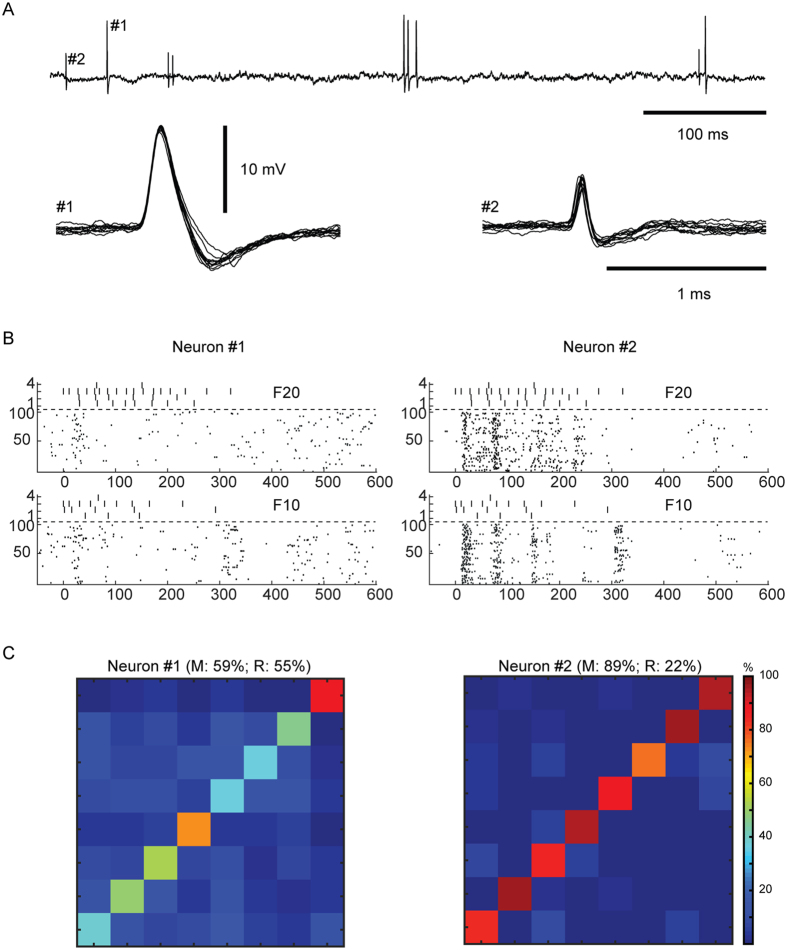
Adjacent neurons could display responses with contrasting temporal patterns. (**A**) Raw trace illustrating the spikes of two neurons recorded in parallel, at two different points in time. Below are shown 10 superimposed spikes from each neuron. Note the much faster spike of neuron #2, suggesting that it may belong to a smaller neuron or interneuron^17^. The amplitudes of the two spikes increased and decreased independently of each other during slight manipulation of the position of the recording electrode (0.3–20 μm of travel with the micromanipulator, not shown), indicating that they were recorded from two separate neurons (see also ref. 25). (**B**) Raster plots for the two neurons for two sample stimulation patterns. Note the large differences in the responses to the same patterns, despite that the neurons most likely were located within a few μm from each other. (**C**) Confusion matrices of the decoding across the stimulation patterns (as in Fig. 2C) for the two neurons.

Whereas the analysis above was limited to the decoding provided by individual neurons, we often noted a mismatch in the temporal response patterns of different neurons to the same stimulation pattern. Together with the tendency of some neurons to preferentially decode certain patterns better than others (Fig. 2E), this hinted at the possibility of complementary decoding between neurons. Such complementariness could allow for co-operative decoding within a small population of neurons, which together might provide a much higher capacity to segregate spatiotemporal patterns of tactile afferent input. To examine the potential for co-operative effects, we first focussed on a subset of our recordings, in which the spikes of two different neurons were recorded at the same time with the same patch pipette^25^ (Fig. 5A, N = 5 pairs) or using two separate adjacent patch pipettes (N = 2 pairs). The temporal spike response patterns could be widely different between two adjacent neurons (Fig. 5B), a phenomenon previously also observed in awake monkeys^26^,^27^. Moreover, the mean decoding and the specific input selectivity (Fig. 5C) could differ substantially between the two neurons in such paired recordings (the range of the differences in the mean decoding was 2–38% units for N = 7 pairs).

**Figure 6:**
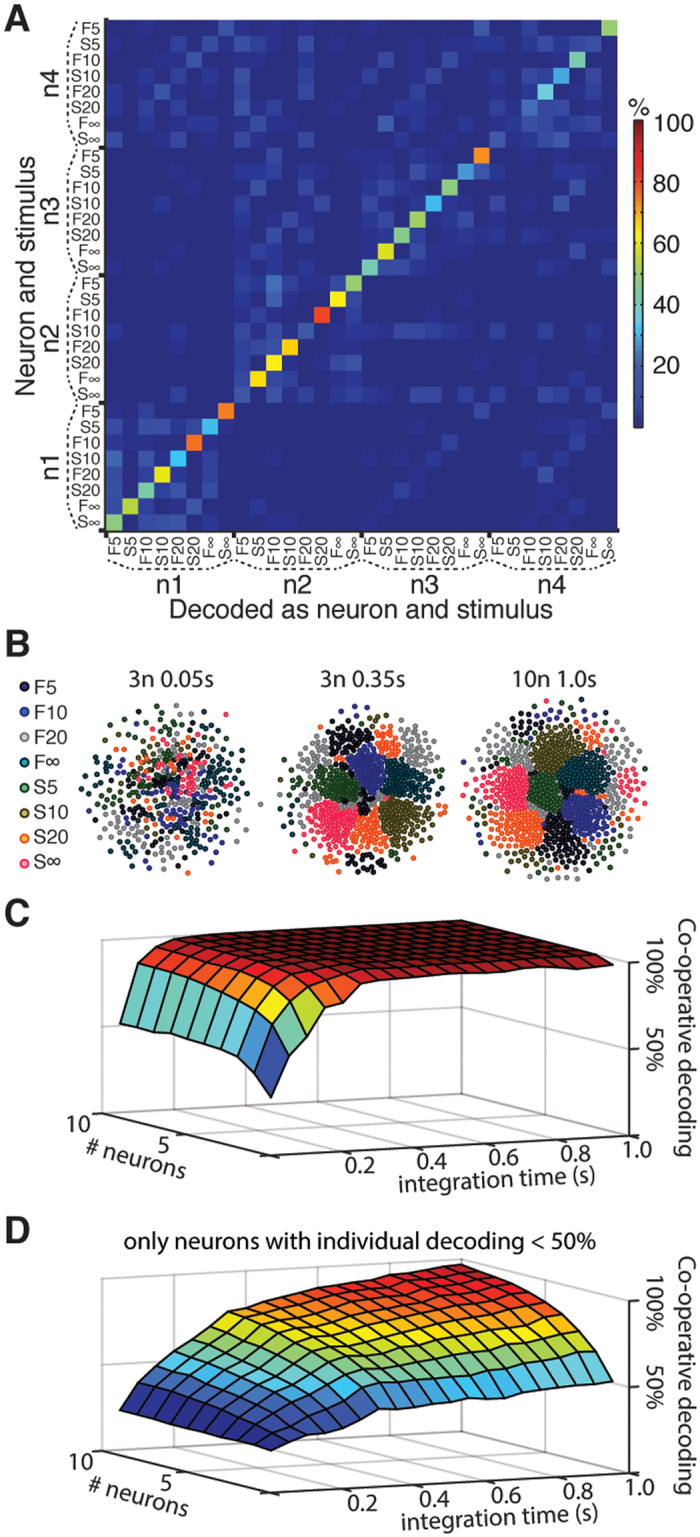
Potential for co-operative decoding in limited neuronal populations. (**A**) Confusion matrix for four high performing neurons, where the temporal patterns of the spike responses to the different stimulation patterns were specific to the neuron. (**B**) The cluster plots show the results of the curvilinear component analysis of the unlabelled spike responses, illustrating the segregation of individual stimulus presentations for the two optimal (with respect to co-operative decoding rate) neurons at 50 ms, the 5 optimal neurons at 350 ms, and the 10 optimal neurons at 1000 ms. The stimulation pattern is color coded as in Fig. 2B. (**C**) 3D plot to illustrate the evolution of the estimated optimal population decoding rate, as evaluated with PCA and kNN, from the repeated presentations of the stimulation patterns and an increasing number of neurons and increasing time. (**D**) The estimated optimal population decoding when the pool of neurons was limited to those with a mean decoding below 5 times chance level.

A cross-neuron—cross-stimulation analysis of some of the top decoding neurons (Fig. 6A) confirmed that the temporal response patterns to the same stimulation pattern could be unique for each neuron. There was also a lack of ‘cross-talk’ between neurons in terms of their individual temporal response patterns to different spatiotemporal stimulation patterns (Fig. 6A). Therefore, we next analysed the potential cooperativity across the entire population of recorded neurons. Cluster analysis indicated that the decoding performance increased with the number of neurons (Fig. 6B), consistent with that each neuron in a population contributes unique information^27^. To further quantify the potential cooperativity effects, we performed a population level PCA (Fig. 6C). We first estimated the optimal population decoding among the entire ensemble of neurons in our dataset as a function of integrated time and number of neurons included. In this case, the analysis of the potential for neuronal cooperativity indicated that saturation up to perfect decoding (>99%) within 350 ms was obtainable with just a few (3) neurons (Fig. 6C). The rapid attainment of a perfect segregation of the input patterns was not surprising given the outstanding performance of each of these neurons individually. However, in this type of analysis, even neurons with poorer performance in terms of mean individual decoding could together reach a near perfect segregation within a short period of time (Fig. 6D). While considering only neurons with individual decoding below 4 times chance level (i.e. mean decoding below 50%, cf. Fig. 2F,G), a very high population level decoding could be obtained within 800 ms provided that there were many (10) neurons contributing to the decoding (Fig. 6D). Although such effects may not be important for the identification of the limited number of input patterns we used, they could still be essential in order to resolve a larger set of stimuli, i.e. when further qualitative details about the properties of the object, or finer shape details, need to be resolved.

### Temporal resolution of the spike responses

The importance of the temporal evolution of the spike responses was further tested by evaluating the decoding obtained assuming a pure rate code (as in the study of Luczak, *et al*.^17^, for example), i.e. where only the total number of spikes evoked by the stimulation pattern was taken into account (Supplementary Fig. S6, a pure rate code corresponds to using Victor Purpura spike distance, VPd, with cost q = 0). In this case, the decoding performance was lost and did not overcome chance level. Across the population, assuming a rate code resulted in significantly worse decoding performance (p = 1.01e-07, non-parametric Wilcoxon signed rank test). However, the temporal dynamics of the spike responses varied substantially between neurons. The optimal cost q was typically between 175 s^−1^ and 50 s^−1^, corresponding to a temporal scale of 12–40 ms (Supplementary Fig. S6).

### Neuronal input segregation depends on the spatiotemporal structure of the input

**Figure 7:**
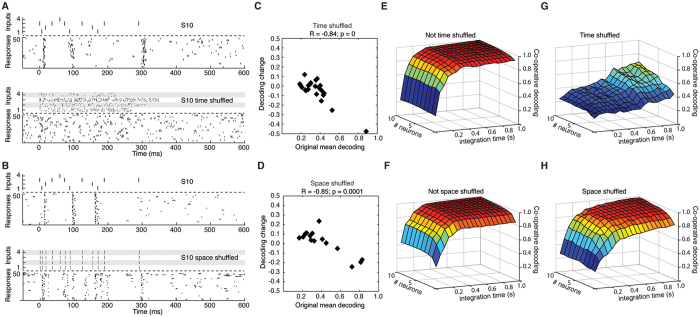
Effect on the decoding performance of temporal and spatial shuffling of the stimulation patterns. (**A**) Example of the effect of temporal shuffling of the S10 stimulation pattern on the spike responses. Top, in the control, the spatiotemporal stimulation pattern was exactly the same from trial to trial. Bottom, with time shuffle, the number of stimulations per channel were held constant but their temporal distribution was shifted at random. (**B**) Similar display for the effect of spatial shuffling of the S10 stimulation pattern. (**C**) Effect of temporal shuffling on the mean decoding across the population of neurons. Note the strong relationship between the level of mean decoding in the control and the loss of decoding in the shuffled condition. (**D**) Effect of spatial shuffling on the mean decoding in another population of neurons, with a similar relationship as in (**C**). (**E**,**F**) Co-operative decoding before shuffling. (**G**,**H**) Co-operative decoding after temporal and spatial shuffling, respectively. (**E–H**), similar display as in Fig. 6C, including up to the 10 best co-operative neurons, sorted before and after shuffling.

We also tested directly whether the spatiotemporal structure of the stimulation patterns was a decisive factor for the decoding performance. First, we shuffled the temporal structure of the stimulation patterns (N = 21 neurons) (Fig. 7A). In neurons with high mean decoding, temporal shuffling substantially decreased the performance (Fig. 7B). In contrast, in neurons with a low mean decoding the temporal shuffling did not degrade the performance and there was a monotonically decreasing correlation between the mean decoding of the neuron and the change in performance resulting from the shuffling (Fig. 7C). We next examined the importance of the spatial structure of the stimulation patterns by maintaining the temporal patterns and the number of spikes in each stimulus channel, but by randomizing the skin site of delivery of the four channels. Although the effects of the spatial shuffling was overall weaker, which is attributable to the comparatively smaller disruptive effect on the stimulation patterns (Fig. 7C), the results were in principle similar (N = 15 neurons) (Fig. 7D). Also the population level decoding was strongly degraded by the temporal shuffling, whereas the effects were weaker but still clear for spatial shuffling (Fig. 7E–H).

## Discussion

The main aim of the present study was to provide a quantitative estimate of how well neocortical neurons can identify the spatiotemporal pattern of skin tactile information, i.e. identifying the ‘what’ component of tactile input received^28^. The decoding accuracy or the information content was in this respect found to be very high in at least some individual neurons. A second main finding was that the same tactile afferent input could be encoded in different temporal response patterns between neurons, which suggest that the brain can channel tactile information through multiple parallel processing networks, possibly focussing on complementary aspects of the input information. To our knowledge, this is the first time the decoding performance of neurons of the somatosensory cortex are investigated using (i) reproducible spatiotemporal patterns of skin tactile afferent activation, (ii) that are delivered in multiple, richly resolvable patterns that resemble natural tactile scenes, i.e. touch of a range of objects. The reproducibility eliminated the need to restrict the analysis to the rate codes of the neuronal responses, as in previous analyses of cortical information^17^,^29^,^30^. Instead, it became possible to focus on the details of the temporal evolution of the spike responses, which turned out to be a domain where a substantial amount of the neuronal information resided.

### High neuronal decoding capacity

Our results indicate very high individual neuronal decoding capacity (up to more than 2.8 bits per neuron, out of 3 bits of maximal information with 8 stimuli) (Fig. 2 and Supplementary Fig. S4) and reveal multiple features that give novel clues to the organization of the sensory processing in neocortical circuitry. We did not find any relationship between decoding performance and recording depth (Fig. 3), which, although a crude measure, suggests that many neuron types participated in the decoding. The high precision decoding was surprising since the internal states of the brain, as in all neurophysiological recordings, were outside our control and varied between trials and undoubtedly represented a major source of noise. During active explorative movement, the neural activity resulting from skin-object interactions can be expected to be much better in register with the brain state, under which circumstance the higher decoding capacity discovered in the intracellular recordings (Fig. 1) is likely to be better reflected also in the spike output. Furthermore, a surprising finding was that the decoding performance of many of the neurons continued to improve up to 700 ms after the termination of the tactile stimulation, which suggests that the tactile input itself affects the state of the processing circuitry with long aftereffects. In this context it might be asked how the anesthesia could affect the decoding. There would seem to be two possible scenarios that are not mutually exclusive. One is a general degradation of network function and therefore decoding. Another one could be that the oscillatory tendencies of the circuitry are facilitated which might even improve decoding, in particular at long latencies.

### Heterogeneity and complementariness of neuronal responses

The heterogeneity between neurons with respect to their selectivity for the different input patterns (Figs 2 and 5) and the complementariness of the specific temporal patterns of their responses (Fig. 6A) suggested that individual neurons may be specialized on particular aspects of the skin-object interactions, i.e. they may represent specific bits and pieces of the information that exist at the population level. These different responsive properties of the individual neurons suggest that they may be connected to partly different neuronal networks. Due to the complementariness of the neuronal responses, even single stimulus presentations were readily resolvable using a limited population of neurons (Fig. 6B–D). Our analysis further indicated that cooperativity between a limited number of neurons was sufficient for a high resolution decoding with rapid identification of stimulation patterns. Cooperativity of decoding amongst neurons have previously been proposed for salamander and macaque retina based on studies of weak pairwise spike firing correlations between pairs of neurons^31^,^32^, but to our knowledge cooperativity based on complementary response profiles to a set of different inputs has not previously been described for cortical neurons processing tactile input. Even though we tested only eight different spatiotemporal patterns of input, in the real world the range of possible natural tactile scenes implies that a huge variety of input patterns need to be resolved from this one part of the skin alone, a situation where the capacity added by the population of neurons is likely to be crucial. Importantly, from this study we naturally cannot say how the integrated information decoding of the neocortex normally works. However, although the brain may well use other methods for segregating the information than those used here, our analysis at least indicates that the neural information is there for the brain to use–it remains to be shown if its neuronal network has the structure and functionality to take advantage of this potential.

### The electrical skin interface, its relation to mechanical stimuli and its potential resolution

Our approach of using haptic stimuli transduced into spatiotemporal patterns of tactile afferent stimulation by a set of neuromorphic artificial sensors was motivated by the aim to eliminate known limitations of mechanical skin sensor activation (see Introduction). I.e. by delivering this input electrically to the primary afferents in local skin sites we wanted to by-pass the step of potentially variable skin sensor activation that occur even with highly controlled mechanical skin stimulation^9^, so that the decoding capacity of cortical neurons could be studied in relative isolation. Just like natural tactile inputs, the input we provided can be expected to be distributed and processed through the neuronal networks in the cuneate nucleus, thalamus and neocortical circuitry before it reached the neurons we recorded from. Hence, the measured decoding is bound to reflect at least in part the inherent processing mechanisms of the brain. Accordingly, in humans, electrical nerve stimulation with a much lower resolution than in the present set of experiments are known to generate sensory impressions that are in part perceived as unnatural but also to generate diversified and meaningful tactile percepts^12^,^13^,^14^,^15^,^16^. We expect our higher-resolution approach to have much higher potential to generate natural sensations, but how much do we know about the relationship between our spatiotemporal patterns and information generated by mechanical skin-object interactions? For each of the four skin sites used, a limited number of skin sensor afferents would be expected to be activated^21^. As the electrical activation is highly reliable^21^, every single stimulation pulse can be expected to activate the local set of skin sensor afferents in synchrony. However, the fact that different sensors have different conduction velocities^33^ results in that this synchrony is broken up already when the input reaches the cuneate nucleus^21^. The fact that slowly and rapidly adapting skin sensors would be expected to be activated to the same extent by the electrical stimuli may first look unnatural, as there are many reviews that tend to stress the different conditions of activation of these types of sensors. However, under the type of dynamic indentation movement we used here, available evidence indicates that there is in principle little difference in activation between slowly and rapidly adaping tactile mechanoreceptors^1^ (Supplementary Fig. S2).

Fundamentally, all skin sensors transduce mechanical skin strain patterns into receptor potentials^34^–under non-static touch conditions, such receptor potentials may be relatively congruent between different types of skin sensors with the same location. The receptor potentials are then translated into patterns of spike output in the primary afferent. In central neurons, the spike generation mechanism is subjected to stochasticity^22^,^23^ and provided that the underlying ion channel mechanisms are related this should apply to skin sensors, too. Indeed, identification of fine details of edge stimuli from recorded spike trains in human tactile afferents was found to work well when the individual spike times was convolved into Gaussian functions with kernel widths up to 8 ms, suggesting this to be a useful upper limit for the temporal precision of the spike generation. Similar findings exist for the rat whisker primary afferents^35^.

The artificial fingertip allowed us to synthesize spatiotemporal patterns of skin sensor activation at quasi-natural rates that follow a natural overall temporal modulation, or ‘envelope’^36^, that the biological skin sensors are known to display under dynamic indentation^1^ (Supplementary Fig. 2). This aspect of our approach is probably important because the circuitry of the cortex can be expected to have experienced many events with similar envelopes of tactile afferent activity and is therefore likely to have adapted its circuitry structure to effectively process variations of that kind of overall activity modulation (as in the auditory and visual cortices^17^,^18^). The expected congruence of the receptor potentials under a dynamic indentation would under natural circumstances tend to drive the tactile afferents spiking to follow similar overall temporal patterns. Given the presence of skin sensor spiking stochasticity, synchronized spiking activity may well occur, as detailed next. For the indentation stimuli used here, with up to 10 spikes per sensor generated during the 300 ms that the stimulus lasted and assuming that 10 local skin afferents were engaged per channel, the spike timing variability expected from the stochasticity implies that a near astronomical number of possible spatiotemporal patterns of primary afferent activation could occur even if exactly the same mechanics of the touch condition could be repeated. This can be considered the space of possible outcomes for that condition. If the same touch condition occurred, but with a slight shift of the mechanical conditions, for example a 0.1 mm change in position across the skin, the space of the possible outcomes would be further widened. With the information being distributed across a high number of tactile afferents, it is still possible for the brain to recognize the tactile experience as belonging to the same kind of object and discriminate it from other experiences. The full space of possible tactile experiences, representing any kind of physical interaction with any kind of possible object, would be very large indeed. Our electrical stimuli, with near synchronous activation of the local skin afferents, would only be required to fall within that huge space of possible outcomes in order to represent a type of input that could theoretically occur under the lifetime of a rat–depending on the actual range of spike timing variability that applies for rat skin tactile afferents, this would hardly seem unlikely.

However, a main caveat is that all the local sensors were driven to the same level of activity. It is questionable whether there exists physically possible skin-object interactions in which all local sensors can be expected to be activated within the same spiking frequency range. This probably makes our stimuli falling short of being able to produce perfectly normally perceived sensations. Rather, our synthetic spatiotemporal patterns of skin afferent activation could produce a sensory experience that would be partly perceived as natural and partly unnatural, i.e. a proper indentation sensation mixed with unnatural sensations such as tingling, where at least rough shape classification may be possible to achieve.

Naturally, whereas the resolvability of the inputs that we provided was sufficiently high to provide quantitative measures of the decoding of the individual neurons, real world mechanical stimuli would be characterized by much higher information content in the population of primary afferent activity^37^,^38^. The logical explanation for this is that as long as the electrical skin interface uses only four channels, and cannot control primary afferents individually, it will never be able to compare with the diversity of information that is potentially possible in the natural system.

### Comparisons with previous related studies

Previous quantitative analyses of the information decoding in somatosensory systems of the brain have focussed on the presence or absence of a vibrotactile input (i.e. 50% chance level^39^) and which body part or whisker an input arises from (i.e. the ‘where’ issue rather than the ‘what’ issue)^40^,^41^. The discrimination of different input patterns in S1 neurons has been explored with sinusoidal stimuli in the whisker system of the rat^42^, where the highest information content was in the order of 0.3 bits over >5 bits of maximum theoretical information, which can be compared with up to 2.86 bits over 3 bits of maximum theoretical information in our study (Supplementary Fig. S4). A likely reason for the rather large discrepancy is that our ‘frozen’ spatiotemporal patterns of primary afferent activation represented more reproducible, and therefore more discriminable, input than mechanical peripheral stimulation, as described in the Introduction.

Electrical primary sensory afferent stimulation has previously also been used to study the responses in the brain induced by cochlear and retinal implants. However, so far, such studies have been limited to analyses of field potentials and cortical topography^43^,^44^, neuronal response latency times^45^ and firing rate^46^, whereas the decoding capacity of individual cortical neurons provided with input from epiretinal implants or other audio/visual implants has not been tested.

### Future developments and applications of the approach

Our findings also have implications for the translational application of biomimetic artificial touch technology in neuroprosthetics and bionic limbs, where the aim is to impart natural sensations to the users^47^,^48^. In a device designed for humans, our approach would equal a very high resolution intraneural interface^13^ accessing multiple terminal nerve fiber branches, innervating adjacent small patches of skin, for individual electrical activation. The activation across the sensors/nerve branches should ideally be delivered in spatiotemporal patterns that respect the realm of possible combinations of sensor activation that would occur in the biological system, i.e. at least capturing the envelope of expected spatiotemporal activation of skin sensors as we aimed for here. This approach may provide the prosthesis with sufficiently rich and reality-like sensory information to make it tunable to the normal brain circuitry processing mode, which combined with brain network plasticity could potentially allow the prosthesis to become a natural member of the mind-body relationship.

In addition to revealing novel features of neocortical neuronal decoding of tactile input, and an approach to analyze neuroprosthetic functionality, our experimental design also offers a potential tool for the analysis of the progression of neurodegenerative disease in animal models of those diseases. The information content of single neurons is likely to be a sensitive indicator of the state of the network of neurons that innervates them–any deterioration in that network can be expected to result in degraded decoding performance. Hence, models of neurological disease, and the effect of drug candidates or training, could be benchmarked with this approach of highly reproducible inputs.

## Methods

### Surgical procedures

For neuronal recordings, adult male Wistar rats (N = 26, weight 300–450 g) were prepared and maintained under anesthesia with a ketamine and xylazine mixture (20:1). This type of anesthesia has no disruptive effect on the order of neuronal recruitment of neocortical neurons in spontaneous brain activity fluctuations (up states, recordings obtained using multielectrode arrays in the rat) as compared to the awake condition, which suggest that the neocortical network may work close to normal^49^, even though the global brain state regulation does not. Anesthesia was induced via an i.p. injection (40 mg/kg of ketamine) and maintenance was administered through an intravenous catheter inserted into the right femoral vein (appx. 5 mg/kg per hour with a continuous infusion). The induction of the ketamine-xylazine anesthesia was preceded isofluorane sedation (2% for 30–60 s). For recording sessions, the level of anesthesia was monitored with an ECoG electrode placed in the vicinity of the recoding area. The ECoG was characterized by the irregular occurrence of sleep spindles, a sign of deep sleep^50^. The level of anesthesia was additionally characterized by an absence of withdrawal reflexes to noxious pinch to the hindpaw. The decision to run the neuronal recording experiments under anesthesia was motivated by that we needed to make sure that the mechanical stability of the brain was consistently high throughout the experiments in order to be able to run the long-term *in vivo* patch clamp recordings necessary to expose the neurons to a sufficient number of stimulus presentations (up to 2000 presentations delivered at 0.5 Hz). All animal experiment procedures in the present study were in accordance with institutional guidelines and were approved in advance by the Local Animal Ethics Committee of Lund, Sweden (permit ID M118–13).

### Recordings

All recordings were made *in vivo*, in the region of the primary somatosensory cortex of the forepaw (Fig. 1C), as estimated by the focus of the local field potentials (between layers III and V) evoked by electrical stimulation of the forepaw and digit 2. The coordinates of this region were 0.0–1.0 mm rostral to bregma and 3.5 and 4.5 mm lateral to –1.0 caudal to bregma and 3.0 and 4.0 mm lateral as defined by a stereotaxic system. Individual neurons were recorded with patch clamp pipettes either in the intracellular, whole cell mode or extracellularly in the loose patch recording mode. Patch clamp pipettes were pulled from borosilicate glass capillaries to 6–15 MOhm using a Sutter Instruments (Novato, CA) P-97 horizontal puller. The composition of the electrolyte solution in the patch pipettes was (in mM) potassium-gluconate (135), HEPES (10), KCl (6.0), Mg-ATP (2), EGTA (10). The solution was titrated to 7.35–7.40 using 1 M KOH. In some cases, the solution also included 1.0% neurobiotin, used for the morphological identification of the neurons. In order to find neurons, recorded signals were continuously monitored on a display and via loudspeakers. During slow advancement of the recording electrode (approximately 0.3 μm per second), all the skin stimulation sites were activated with one pulse per second, and any neuron thus encountered were typically recorded from. In some experiments we used two pipette electrodes to obtain dual recordings, and in some cases dual recordings were obtained from a single electrode. All data was digitized at 100 kHz using CED 1401 mk2 hardware and Spike2 software (Cambridge Electronics Devices, CED, Cambridge, UK). The recording depth from the surface of the brain was annotated. For identification of neuron identity, made in a subset of our recordings, in addition to depth we used direct morphological identification, using post-mortem histological identification in a confocal microscope (Nikon A1R+), as a pyramidal cell or a non-pyramid cell combined with approximate layer identity using anatomically verified recording depth (using measurements of the distance from the pial surface) (N = 15) (Fig. 1D), and the nature of the firing during spontaneous activity (i.e. if the neuron was fast-spiking, bursting and what duration and intensity of bursts the neuron displayed). After the recording session, the animals were sacrificed and perfused with 4% paraformaldehyde. In the post-mortem histological processing, Alexa488 fluourofor conjugated with streptavidin (Molecular Probes Inc.) was used to stain the recorded neurons.

### Stimulation

Four pairs of intracutaneous needle electrodes (channels 1–4) were inserted percutaneously into predetermined sites in the skin on the volar side of the second digit of the forepaw and constituted the electrical interface used for stimulus delivery (Fig. 1B). The interneedle distance in each pair of bipolar electrodes was 2–3 mm. With this type of approach, the threshold for activating individual primary sensory afferents is in the order of 0.2 mA^21^,^51^. Here, for each skin site, the elementary stimulation pulse was set to an intensity of 0.50 mA and stimulation pulse duration of 0.14 ms (DS3 Isolated Stimulator, Digitimer, UK), which should be well below the 4 times the threshold intensity where A-delta fibers start to become recruited (peak activation requires 6–10 times threshold intensity)^20^.

### Neocortical neuron recording with spatiotemporal patterns of skin activation

The experimental data was neocortical neuronal responses to eight predefined spatiotemporal patterns of skin site activation, patterns that were composed of multiple electrical stimulation pulses to the four skin sites used (see ‘*Generation of spatiotemporal spike stimulation patterns*’ below). The eight spatiotemporal stimulation patterns were delivered in a pre-defined random (pseudo-random) order, where the stimulation patterns lasted for less than 340 ms and the consecutive deliveries of the stimulation patterns was separated by 1.8 s in order to allow a relaxation of the cortical activity induced by the stimulation. Only neurons that could be recorded for at least 120 trials of stimulation pattern presentations were included in the analysis. However, most neurons could be recorded for at least 350–800 trials, i.e. up to 100 presentations of each of the eight predefined stimulation patterns.

### Generation of spatiotemporal spike stimulation patterns

In order to achieve as realistic spatiotemporal patterns of electrical skin stimulation as possible, while preserving the aim of high reproducibility of the patterns, we used an artificial fingertip equipped with a set of four neuromorphic sensors to generate the spatiotemporal patterns of skin activation to be used in the electrical interface with the rat skin. The four electrode pairs of the interface were 1-to-1 connected to the four neuromorphic sensors of the artificial fingertip, and the neuromorphic sensors generated spiking output that was used as a trigger for the elementary electrical stimulation pulses of the interface with the animal. To induce sensor activation patterns that could occur in active touch, a scotch-yoke mechanism (Fig. 1A) was used to mechanically indent the sensorized fingertip against probes of four different shapes (Supplementary Fig. S1A–C). The core element of the sensorized fingertip was a Micro Electro Mechanical System (MEMS) sensor with 4 transducing piezoresistors implanted at the base of a cross-shaped structure^52^ (Supplementary Fig. S1). The MEMS was packaged with polymeric compliant material (Dragon Skin, Smooth-On, USA). MEMS data were sampled at 380 Hz per sensor output by a 24-bit Analog to Digital Converter (ADS1258, Texas Instruments, USA) integrated in the fingertip, and acquired via SPI by a Field Programmable Gate Array (Cyclone II FPGA, Altera, USA). The FPGA streamed the acquired information via Ethernet to a PC for implementation of an artificial mechanoreceptor model.

Per each stimulation channel *x* (with *x* spanning from channel 1 to channel 4, Fig.1E and Supplementary Fig. S1), in our neuromorphic artificial touch system^53^ we used customized implementations of the Izhikevich spiking neuron model^54^ to emulate 2 artificial mechanoreceptor types, mimicking to some extent slowly (labelled S model) and fast (labelled F model) adapting receptors (see Supplementary Fig. S2 for comparison with electrophysiological literature under similar experimental conditions). The difference between the implementations of the two artificial mechanoreceptor types relied in the input to the model. In the S model, opponent sensor channel pairs *Sx*+ and *Sx−* (see Supplementary Fig. S1A for labelling) were subtracted to generate a signal (*S*_*x*_, see Eq. 1) that was normalized and half-rectified to produce (Eq. 2; for values of parameters, see Supplementary Table S1) the external input *I*_*x*_ (Eq. 3). The S model was a multi-channel implementation of a previous artificial receptor that had been used to elicit tactile percept in human subjects with single-channel intrafascicular neural stimulation^12^. In addition to the S model, we introduced the F model in which the external input was given by the time derivative of *I*_*x*_ (Supplementary Fig. S1A and Eq. 4) to achieve a faster sensitivity to stimulus dynamic changes. Note that the F model does not fire under a sustained indentation, however this was not appreciable in the present experimental data since the protocol did not include static post-indentation phases due to the continuous motion of the scotch-yoke mechanism (Supplementary Fig. S1C). In both models the adaptation variable *u*_*x*_ evolution followed Eq. 5 and if the membrane potential *v*_*x*_ reached the threshold value of 30 mV one spike was released and both the membrane potential and the adaptation variable were reset according to Eq. 6. In dimensioning the parameters for the Izhikevich spiking neuron model^54^, we used the set of coefficients inducing regular spiking with weak adaptation^53^. Whenever the membrane potential *v*_*x*_ reached the threshold level (Eq. 6), a spike was triggered, *v*_*x*_ was set to a reset value *c* and *u* was increased of a fixed value *d*.


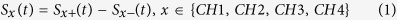



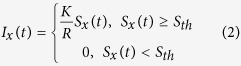



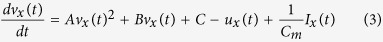



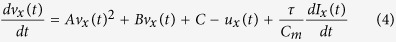



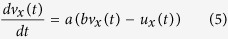



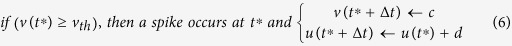


Per each channel *x* (CH1 to CH4), the computed binary response constituted the output of the neuromorphic artificial touch system. Such spike sequences were broadcasted by TCP communication to successive services and graphical user interface (Labview, NI, USA), that was responsible to generate the trigger per each channel, via a BNC connection to a current stimulator (Digitimer unit, see above) allocated for each electrical channel interfaced to the skin (Supplementary Fig. S1A and Fig. 1B).

### Decoding based on sensors outputs and selection of spatiotemporal stimulation patterns

In order to evaluate whether the degree of segregation of the spatiotemporal patterns of sensor output was sufficient to deliver resolvable information to the somatosensory system, we first implemented a repeated (N = 100 per stimulation pattern) delivery of the mechanical stimulation to the biomimetic fingertip. Such stimulation was carried out via the 4 different probes and with the 2 different artificial mechanoreceptor models, yielding a total set of 8 stimulation patterns (Supplementary Fig. S1).

Neurophysiological considerations^55^ suggest that the nervous system appreciates the fine temporal differences between the spike trains arriving from different channels. These differences can be quantified with the Victor Purpura (VP) metrics^56^. VP metrics defines the distance between two spike trains as the minimum cost of transforming one into the other by following two operations (see Supplementary Fig. S3): adding/removing a spike (cost:1) and shifting a spike by an interval Δt (cost = q*Δt). Note that the shift cost parameter *q* identifies the relevant timescale for the comparison^56^. In order to introduce a distance between multi-channel spike trains, we computed the sum over the four channels of the VP distances measured on a channel by channel basis between spike train pairs (i.e., defining a 4-dimensional *l*^1^ norm based on VP-distance, which can also be termed a 4-dimensional Manhattan VP-distance). We measured this distance across all presentations and all stimuli (Supplementary Fig. S1F). To test whether it was possible to segregate the 8 spatiotemporal stimulation patterns according to these distances we applied a k nearest neighbors (kNN) clustering decoding procedure^57^, since spike trains with small distances, i.e. neighboring responses, are likely to be elicited by the same stimulation whereas spike trains with large distances are likely to be elicited by different stimulations. Specifically the kNN was implemented with k = 20 (out of 100 stimulus presentations) and using half of the trials picked at random as training set. We evaluated decoding performance computing the mutual information between presented and predicted stimulus at each trial^58^, as depicted by the confusion matrix that represents (column-wise) the computed decoding (correct responses in the diagonal) per each presented stimulus (labelled row by row) (Supplementary Fig. S1G). Information was computed with the Information Breakdown Toolbox in MATLAB (MathWorks). We evaluated information bias combining quadratic estimation and bootstrap subtraction^59^.

After the evaluation of the degree to which the sensor output patterns obtained from the repeated (N = 100) mechanical stimulation of the biomimetic fingertip could be segregated, we selected (Supplementary Fig. S1D) a representative spatiotemporal pattern of sensor spike output for each probe/receptor model condition to be systematically delivered in the neurophysiological experiments. Per each stimulus condition, among the spatiotemporal patterns that were below the threshold level (VPd < 1.5, Supplementary Fig. S1E), the sensor output pattern to be used as the representation of that stimulus condition was selected at random.

### Decoding based on single neuron spike responses and principal component analysis (PCA)

The spiking responses of single cortical neurons to the different spatiotemporal input stimuli were analysed using PCA. This analysis answered the question by which precision the spiking response could be used to distinguish one stimulation pattern from the others. We decoded the stimuli from the spiking patterns of single cortical neurons using an improved and simplified version of a method^60^ based on the decomposition of the temporal profiles of the evoked spike responses into Principal Components (PCs):

- We computed the mean firing rate over time in the response to each stimulus presentation.
- We converted the spike trains evoked by each stimulus presentation into continuous functions by convolving them with an exponential kernel with a characteristic time of 5 ms.
- We computed the average of the resulting functions for each stimulation pattern to obtain a template of the average temporal evolution of the response to each stimulus.
- To highlight the differences in the temporal profile we z-scored the average responses.
-   We extracted the principal components (PCs) of the z-scored average responses evoked by the different stimulation patterns and we computed for each individual response the score relative to each PC, that is, the scalar product between the response temporal vector and the PC temporal vector.
- The decoding was performed using the mean firing rate and the scores on each of the first 7 PCs for each individual response. The average intensity and the temporal profile of the individual response hence determined the location of that response in this 8-dimensional space;
- In order to decode the stimuli from the response patterns we used the kNN classification procedure as previously described^60^. As stated in the previous point, the response to each stimulus presentation was represented as a point in a space with 8 dimensions. Half of these points were selected as random as training set. For each trial belonging to the test set we identified the closest 9 trials in the training set with an Euclidean distance in the 8-dimensions space. The trial was then classified as elicited by the same stimulus that elicited the relative majority of the 9 neighbors. We performed 40 iterations of the decoding, each with a different training set, and we averaged the fraction of correctly decoded trials in each iteration to get the decoding average of the neuron. We also computed and averaged over all iterations the confusion matrix information (see previous subset).

The analysis of the spike responses using PCA always included the first 600 ms of the evoked responses unless otherwise indicated.

### Relationship between neuron depth and decoding

The relationship between the depth of the recorded neurons and the decoding performance was tested using Pearson linear correlation analysis and Kruskal Wallis test on 200 micrometer “layers” of the recording depths.

### Analysis of intracellular responses–general

In order to analyse the synaptic inputs to the cortical neurons, intracellular data from whole cell recordings was obtained (Fig. 1E–I). Using a mild hyperpolarizing current injection, the neurons were prevented from spiking and the intracellular membrane potential driven by the synaptic inputs could be analysed in isolation. The eight different stimulation patterns were presented repeatedly, in random order, to obtain multiple membrane potential responses for each stimulation pattern. As the intracellular recordings did not always last for the entire duration of the 50 times 8 stimulus presentations, the number of repetitions varied between the recordings and was 38 ± 10 repetitions per stimulation pattern and neuron.

### Analysis of intracellular responses–PCA

The PCA of the intracellular responses was made in the same way as the PCA of the spike responses described above, except steps (i) & (ii) as the raw intracellular traces already corresponded to the continuous functions. First, to remove high-frequency fluctuations the traces were low-pass filtered (with a 2 ms wide moving average) and re-sampled to 1000 Hz. Secondly, to remove slow shifts in DC offset, raw traces were high-pass filtered (with a cut-off frequency of 0.5 Hz using a first order Butterworth high-pass filter in MATLAB). Then the PCA was performed as described above.

### Analysis of intracellular responses–pairwise comparisons

The raw intracellular traces were low-pass filtered as described above and divided into 1 ms time bins representing averages over 100 samples (as we used a sampling frequency of 100 kHz) each (‘bin averages’). The membrane potential responses obtained on repeated presentation of each stimulation pattern provided a distribution of the bin averages for each time bin. 10% of the values from this distribution were excluded as outliers in order to minimize the influence from major spontaneous changes in membrane potential. The resulting distributions without outliers were subsequently used to compare the responses of different stimulation patterns in order to see if there were any discernible differences. The Welch student’s t-test was used to evaluate the individual bin averages using the test statistics as a dissimilarity measure between the distributions. The dissimilarity measures were in turn divided into two distributions, one containing the measures of a 300 ms pre-stimulus interval and one 300 ms interval with the measures from the response evoked by the stimulus. These two distributions were used to evaluate whether the difference during the stimulation was larger than that during spontaneous activity. As the p-value in this case is dependent on the number of observations included, this number was kept constant. If the one-sided two-sample Kolmogorov-Smirnov test could reject the null hypothesis that the p-values of the evoked activity were similar or larger than those from the spontaneous activity, we considered a difference to exist between the responses of the two stimulation patterns. Since there were eight stimulation patterns, for each neuron there were 28 comparisons that could be made.

### Estimation of optimal population decoding

In order to analyse the additional decoding capacity that could be provided by a population of neurons, we extended the principal component analysis above. The aim of this analysis was to find the best possible decoding that could be achieved by taking the combined responses of multiple neurons into account. To illustrate how this decoding depended on the duration of the time window and the number of neurons taken into account, this analysis aimed at finding the best possible combination of neurons for each time window and each neuron added to the decoding. Due to the very high number of permutations that would have to be investigated in order to find the combination of neurons with the optimal population decoding performance, the selection process had to be approximated. For each time window, in order to find the combination of N neurons with approximately the best decoding performance, the optimal combination of N-1 neurons was extended with the neuron that had the largest positive influence upon the decoding performance when added to the N-1 neurons. This procedure was repeated for each time window (from 50 ms up to 600 ms in 50 ms steps) increasing the number of neurons from N = 1 to 10.

### Cluster analysis using curvilinear component analysis

The convolved traces (see point (i) under ‘*Decoding based on single neuron spike responses and principal component analysis*’) were also used to investigate whether the responses could be segregated using unlabeled methods that had only access to the responses but not the stimulation pattern that evoked the response. Unlike the kNN classification approach above, where the average response evoked by a specific stimulation pattern was used to compute the PCs, in the unlabeled setting the PCs were computed from the individual traces without knowledge of the ID of the stimulation patterns. For illustration purposes, the PC projections data were embedded into a 2D plane using curvilinear component analysis^61^,^62^ where each individual response was represented by a dot (Fig. 2B).

### Time and space shuffling operation and analysis

In order to explore whether the spatiotemporal patterns of the inputs was necessary for the neuronal input segregation, we also investigated for a subset of the neurons the neural responses to spatial and temporal shuffling, respectively, of the stimulation patterns. For the temporal shuffle, the number of stimuli for each channel was the same as in the normal pattern but the temporal pattern of the delivery was varied at random from trial to trial. For the spatial shuffle, the temporal pattern of each channel was preserved but the output of each channel was delivered to a random skin site.

## Additional Information

**How to cite this article**: Oddo, C. M. *et al*. Artificial spatiotemporal touch inputs reveal complementary decoding in neocortical neurons. *Sci. Rep.* **7**, 45898; doi: 10.1038/srep45898 (2017).

**Publisher's note:** Springer Nature remains neutral with regard to jurisdictional claims in published maps and institutional affiliations.

## Supplementary Material

Supplementary Information
