# Supplementary material for: Artificial spatiotemporal touch inputs reveal complementary decoding in neocortical neurons
Source: Sci Rep. 2017 Apr 4;7:45898. doi: 10.1038/srep45898 (PMC5379202; doi:10.1038/srep45898)
Supplement: Supplementary Information [file srep45898-s1.pdf]

Supplementary Information

Artificial spatiotemporal touch inputs reveal complementary decoding in neocortical neurons

Authors: Calogero M. Oddo, Alberto Mazzoni, Anton Spanne, Jonas M.D. Enander, Hannes Mogensen, Fredrik Bengtsson, Domenico Camboni, Silvestro Micera, Henrik Jörntell

**K**	**A**	**B**	**C**	**Cm**	**R**	**A**	**b**	**c**	**d**	$\mathbf{v_{th}}$
15,000	$\frac{0.04}{sV}$	$\frac{5}{s}$	$\frac{140V}{s}$	$1F$	1Ω	$\frac{0.02}{s}$	$\frac{0.2}{s}$	$-65mV$	$8mV$	30mV

Supplementary Table S1. Parameters of Equations 2-6.

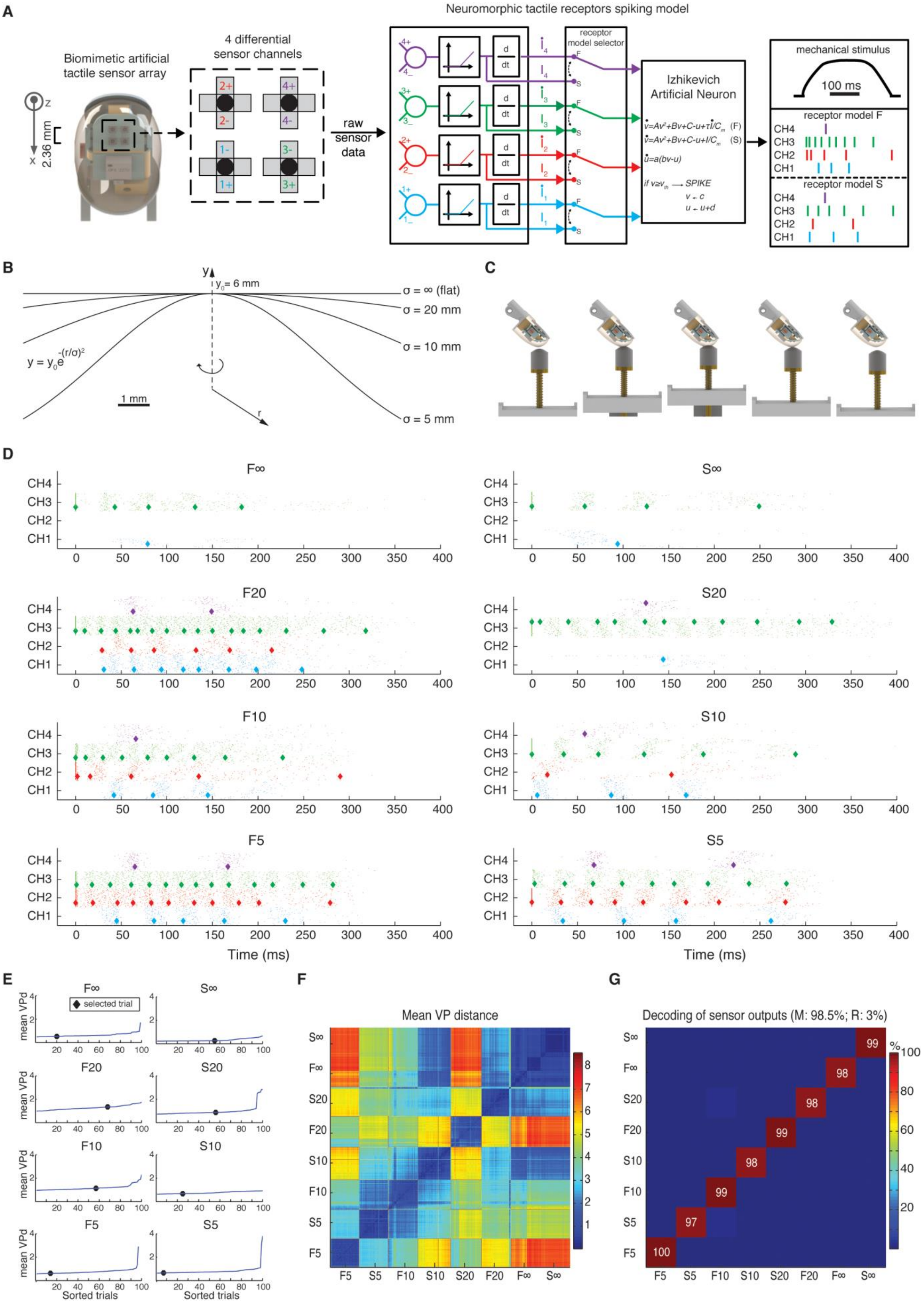

Supplementary Figure S1. The artificial fingertip and the stimulation patterns. (**A**)

Structure of artificial fingertip and properties of its sensors with spike encoding resulting from

implementation of Izhikevich artificial neuron model to four differential output pairs [1]. Two receptor models were implemented by feeding the artificial neuron with their sensor outputs directly or with their time derivative. (**B**) Diagram illustrating the parameters of the four different shapes used for mechanical stimulation. (**C**) Sequence of fingertip movement activating the mechanotransduction of the artificial tactile sensors. (**D**) Raster plots of the artificial sensor output (in the two different skin receptor models) to 100 repetitions of the mechanical stimulus delivery of each of the four shape stimuli. The selected patterns of sensor activation, which were later delivered electrically to the skin of the second digit of the rat, are indicated in stronger color/linethickness. (**E**) Degree of centrality of the selected stimulation patterns with respect to all the 100 repetitions of the mechanical stimulus delivery. (**F**) 4-dimensional Manhattan Victor Purpura distances across all presentations and all stimulation patterns (see Materials and Methods). (**G**) Segregation of the stimuli under the eight different conditions (four probes and two receptor models) illustrated in a confusion matrix of the sensor output decoding over all sessions. 'M' indicates the mean decoding, and 'R' the range of decoding (max-min). All values are obtained as the average over 50 decoding procedures with different training sets.

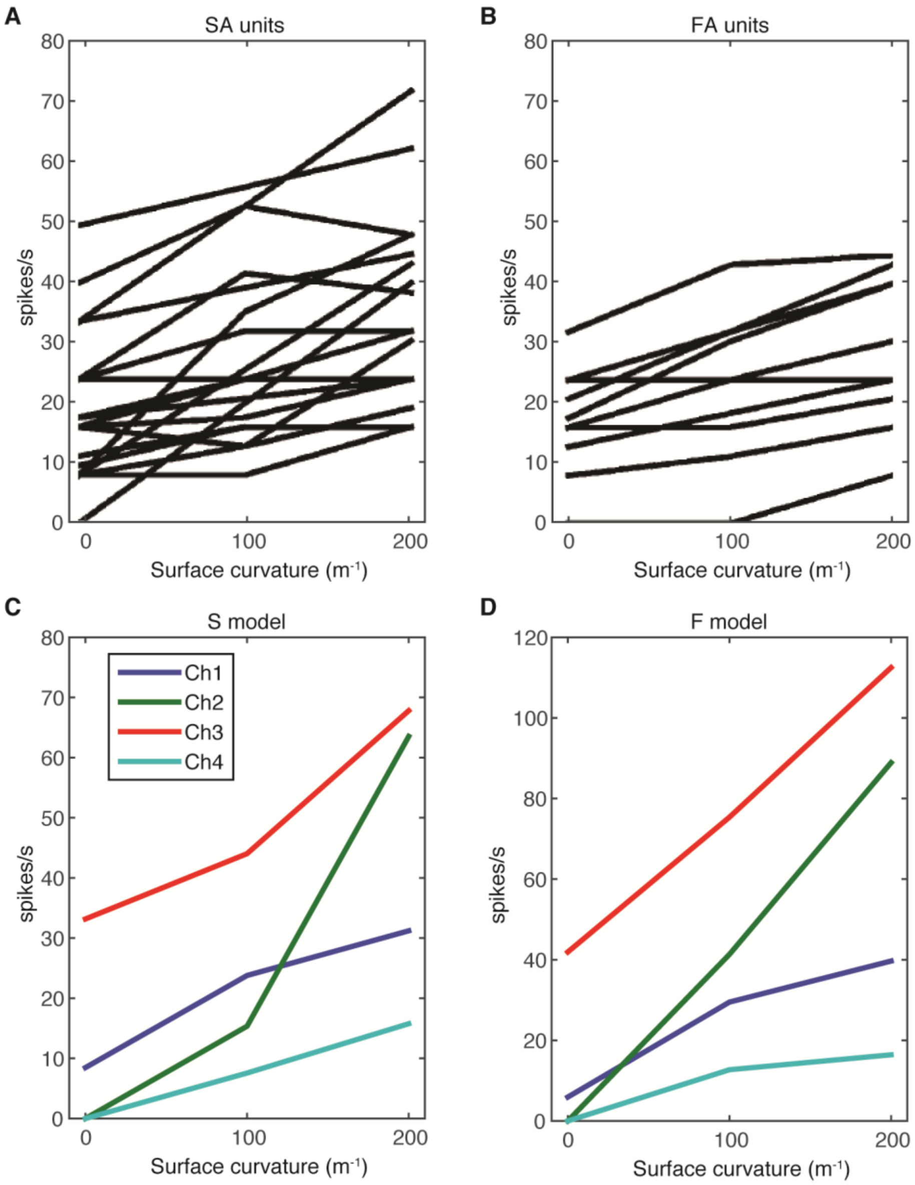

Supplementary Figure S2. The sensors of the artificial fingertip have similar activation properties as human tactile afferents. (**A,B**) Firing frequencies of rapidly adapting (A) and slowly adapting (B) human tactile afferents to indentation of the fingertip with a set of probes with different curvatures. Each line represents the firing frequencies of a single afferent across the curvatures. The data displays are modified from Figs 4A,C of Jenmalm, et al. [2] and represent the spike data from the dynamic phase of the indentation, lasting 125 ms, for the afferents with a positive correlation between their spike responses and curvature, which was the most common correlation. (**C,D**) For the neuromorphic sensors, the corresponding

relationships between the sensor spike outputs and probe curvature for the first 125 ms of indentation, i.e. similar stimulus conditions as in A and B. Each line represents the output for each sensor channel of the artificial fingertip. The data are from the specific sensor spiking output patterns selected (Supplementary Fig. S1D,E) to be delivered by electrical stimulation to the four skin sites of the second digit of the rat (Fig. 1E). Because of the overall similarities in tuning between individual biological and neuromorphic sensors with respect to the different kinds of probes used, we conclude that the synthetic patterns we generated by active touch with the artificial fingertip are likely to have at least overall similarities with patterns of skin sensor activation that could be evoked under the natural condition of active touch.

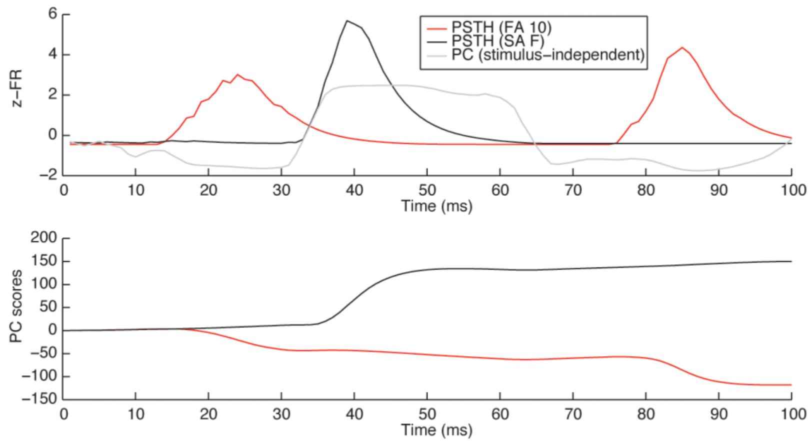

Supplementary Figure S3. Method of PCA illustrated. Smoothed curves (top) are convoluted exponential kernels of the spike responses to a stimulus presentation (here represented by the average of all stimulus presentations for clarity) illustrated for two different stimulation patterns. The binary curve in grey indicates the principal component against which the obtained curves are compared. As the black convolved trace is captured by the corresponding positive deflections of the principal component, this pattern is given a positive scalar value (bottom diagram) for this principal component. In contrast, the red convolved trace is negatively correlated with the negative deflection in the principal component and is hence given a negative scalar value for this principal component (bottom diagram). Same neuron as in Fig. 2A-C.

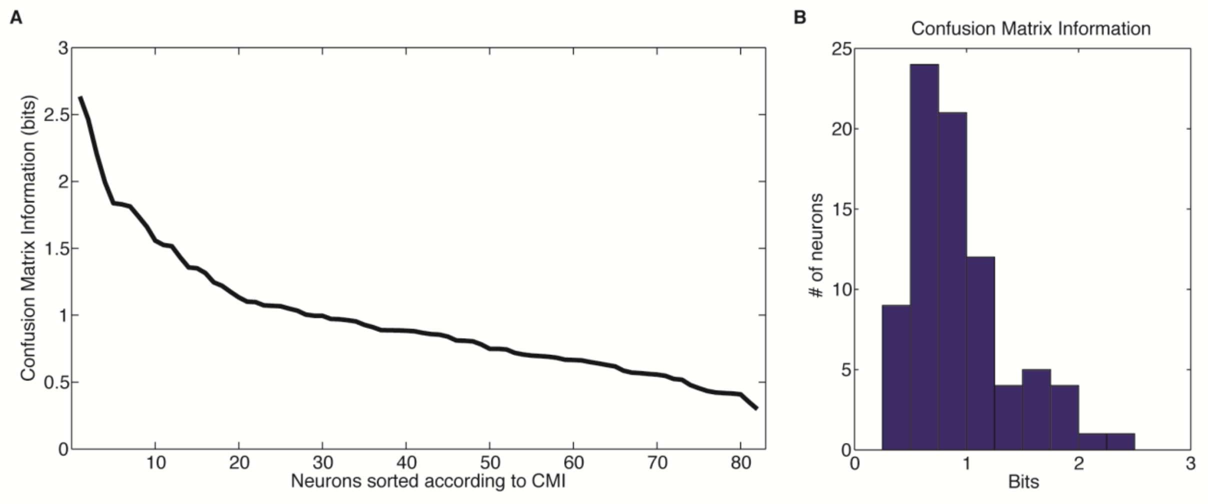

Supplementary Figure S4. Confusion matrix information for neuronal spike response decoding. (A) Similar display as in Fig. 2F but here reporting confusion matrix information rather than decoding performance. (**B**) Histogram of the distribution of the confusion matrix information across the population of neurons (similar display as in Fig. 2G, top).

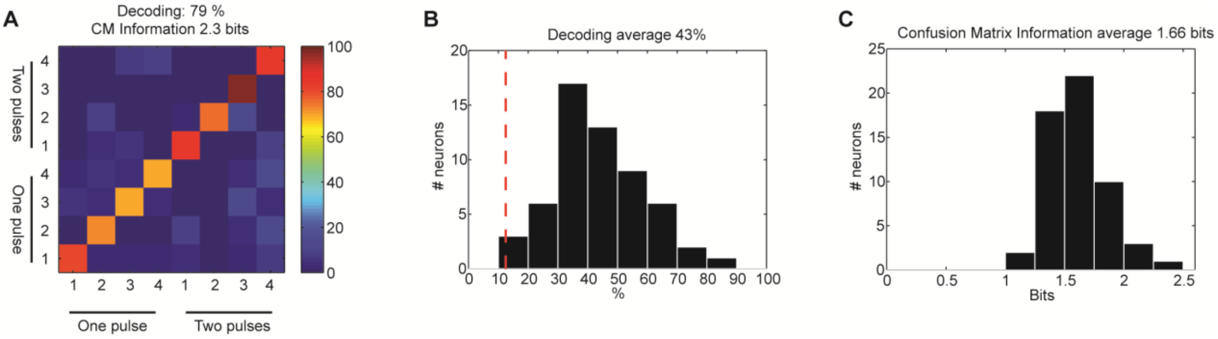

Supplementary Figure S5. Sensitivity to single and double pulse stimulation delivered to individual skin input channels. (**A**) Confusion matrix of a highly performing neuron. Numbers indicate the specific skin input channel (Fig. 1B). Single or double pulse stimulation is also indicated. (**B**) Distribution of decoding performance across 57 neurons. (**C**) Distribution of confusion matrix information across the same set of neurons.

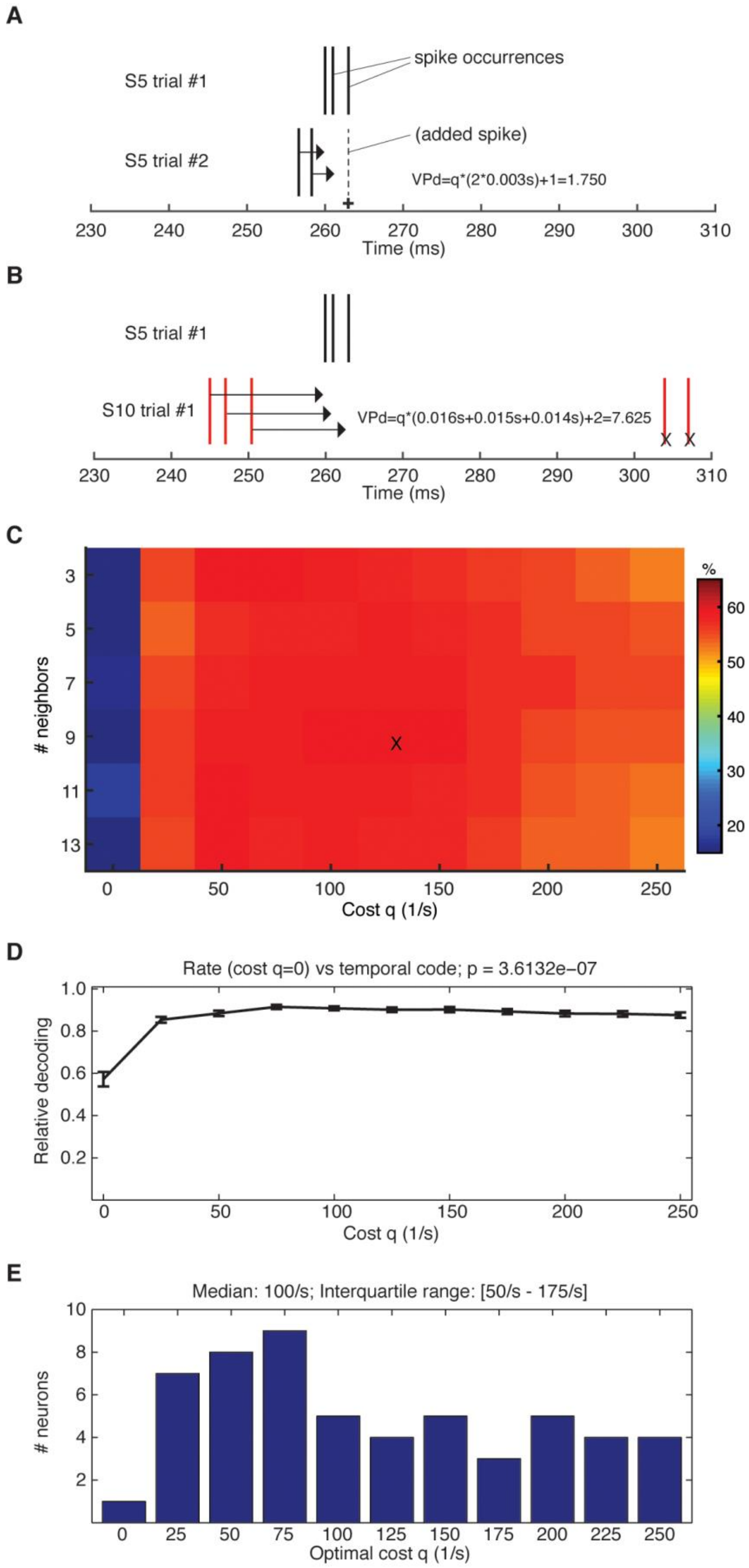

Supplementary Figure S6. Victor Purpura distance calculations for neuronal spike responses. (**A,B**) Illustration of the method, shown by example for a portion of two sample spike trains obtained from the same stimulation pattern (**A**) and for two spike trains obtained

using different stimulation patterns (**B**). Victor Purpura distance is defined as the optimal sequence of operations minimizing the cost of moving spikes (with q cost per unit of time) and deleting or adding spikes (with 1 cost per addition or deletion) with the purpose of making two spike trains identical [3]. (**C**) Left, VPd plot from a sample neuron illustrating the decoding obtained as a function of the cost q and the number of neighboring spikes considered. The optimal decoding was obtained at the point indicated by 'X'. Cost q=0 equals analysing the spike trains as rate codes. Same neuron as in Fig. 2A-C. (**D**) Decoding performance across the population plotted as a function of VPd cost for 55 neurons, which passed the threshold of 15% of mean decoding for the VPd. For each neuron, performance is expressed as the fraction of the performance obtained at the optimal timescale for the neuron. Error bars represent standard error of the mean. The difference between the performance of the rate code (q=0) and the temporal code (q=25/s, the worst performance of temporal codes) is significant with p=1.01e-07 according to the non-parametric Wilcoxon Signed Rank Test. (**E**) Distribution of optimal cost across the same population of neurons. The median value is q=100/s (corresponding to a relevant timescale of 2/q=20 ms) and interquartile range goes from q=50/s to q=175/s (from 40 to 12 ms).

REFERENCES

1 Rongala, U. B., Mazzoni, A. & Oddo, C. M. Neuromorphic Artificial Touch for Categorization of Naturalistic Textures. *IEEE Transactions on Neural Networks and Learning Systems* (2015).

2 Jenmalm, P., Birznieks, I., Goodwin, A. W. & Johansson, R. S. Influence of object shape on responses of human tactile afferents under conditions characteristic of manipulation. *Eur J Neurosci* **18**, 164-176 (2003).

3 Victor, J. D. & Purpura, K. P. Nature and precision of temporal coding in visual cortex: a metric-space analysis. *J Neurophysiol* **76**, 1310-1326 (1996).
